# Compendium of Clinical Variant Classification for 2,246 Unique *ABCA4* Variants to Clarify Variant Pathogenicity in Stargardt Disease Using a Modified ACMG/AMP Framework

**DOI:** 10.1155/2023/6815504

**Published:** 2023-12-26

**Authors:** Stéphanie S. Cornelis, Miriam Bauwens, Lonneke Haer-Wigman, Marieke De Bruyne, Madhulatha Pantrangi, Elfride De Baere, Robert B. Hufnagel, Claire-Marie Dhaenens, Frans P. M. Cremers

**Affiliations:** ^1^Department of Human Genetics, Radboud University Medical Center, Nijmegen, Netherlands; ^2^Center for Medical Genetics Ghent, Ghent University Hospital, Ghent, Belgium; ^3^Department of Biomolecular Medicine, Ghent University, Ghent, Belgium; ^4^Department of Pathology and Cell Biology, Columbia University Irving Medical Center, New York, NY, USA; ^5^PreventionGenetics (a Division of Exact Sciences), Marshfield, WI, USA; ^6^Ophthalmic Genetics and Visual Function Branch, National Eye Institute, National Institutes of Health, Bethesda, MD, USA; ^7^Univ. Lille, Inserm, CHU Lille, U1172-LilNCog-Lille Neuroscience & Cognition, F-59000 Lille, France

## Abstract

Biallelic variants in *ABCA4* cause Stargardt disease (STGD1), the most frequent heritable macular disease. Determination of the pathogenicity of variants in *ABCA4* proves to be difficult due to (1) the high number of benign and pathogenic variants in the gene; (2) the presence of many rare *ABCA4* variants; (3) the presence of complex alleles for which phasing data are absent; (4) the extensive variable expressivity of this disease and (5) reduced penetrance of hypomorphic variants. Therefore, the classification of many variants in *ABCA4* is currently of uncertain significance. Here, we complemented the *ABCA4* Leiden Open Variation Database (LOVD) with data from ~11,000 probands with *ABCA4*-associated inherited retinal diseases from literature up to the end of 2020. We carefully adapted the ACMG/AMP classifications to *ABCA4* incorporating ClinGen recommendations and assigned these classifications to all 2,246 unique variants from the *ABCA4* LOVD to increase the knowledge of pathogenicity. In total, 1,248 variants were categorized with a likely pathogenic or pathogenic classification, whereas 194 variants were categorized with a likely benign or benign classification. This uniform and improved structured reclassification, incorporating the largest dataset of *ABCA4*-associated retinopathy cases so far, will improve both the diagnosis as well as genetic counselling for individuals with *ABCA4*-associated retinopathy.

## 1. Introduction

Biallelic variants in *ABCA4* are the cause of Stargardt disease (STGD1) [1], which is the most frequent heritable macular degeneration [2, 3]. It is estimated to affect between 1 : 6,500 and 1 : 20,000 people [4–7]. The broad clinical spectrum includes classical STGD1 (onset between 10 and 40 years), cone-rod dystrophy (CRD) (onset before 10 years), and late-onset STGD1 (onset after 40 years) [8, 9]. *ABCA4*-associated retinopathy (*ABCA4-*AR) is, therefore, sometimes used as an umbrella term for all retinal phenotypes associated with *ABCA4*. Due to the recessive nature of the disease, the different levels of severity of variants, and the large allelic heterogeneity, it is currently challenging to genetically diagnose individuals with *ABCA4-*AR [10].

Furthermore, in determining the pathogenicity of an *ABCA4* variant, multiple other factors should be considered. Several missense and synonymous variants are known to cause splicing defects in *ABCA4* [11–13]; therefore, missense variants at positions that are not conserved and synonymous variants cannot simply be dismissed as likely benign. In addition, reduced penetrance has been reported for multiple hypomorphic variants in *ABCA4* [14, 15] meaning that variants with a relatively high allele frequency can still be pathogenic. One might expect that when two *ABCA4* variants are detected in a person with a STGD1-like phenotype, these must be biallelic and causal. However, it is not uncommon to find variants to be *in cis* in *ABCA4*, and multiple complex alleles have been described [16–18]. Several studies indicate that in cases with a single variant in *ABCA4* or a single complex *ABCA4* allele, the disease-causing variant(s) can be found in other genes [19–21]. Moreover, the effect of severity on protein function varies enormously among *ABCA4* pathogenic variants. Two deleterious loss of function or null alleles can cause legal blindness before the age of 10 [22–25], whereas mild variants usually only cause disease when present *in trans* with a null or severe allele. These mild variants are associated with foveal sparing [14, 26], resulting in a late age at onset. Furthermore, population statistics and family studies revealed reduced penetrance for some of these variants [15]. The variant c.5603A>T (p.(Asn1868Ile)) has been shown to cause visual impairment in ~5% of individuals when *in trans* with a deleterious allele [14, 26], rendering it a clear hypomorphic variant. This further illustrates the complexity of *ABCA4*-AR and the difficulty of classifying *ABCA4* variants.

Consequently, many individuals with *ABCA4*-AR are currently not genetically diagnosed, as only one pathogenic variant or allele or biallelic variants of uncertain significance have been identified. For these individuals, it is important to know whether the variants they have are pathogenic. Currently, determining the pathogenicity of *ABCA4* variants is crucial as many clinical trials for gene-specific therapies require individuals to have biallelic pathogenic alleles to be eligible for participation. Moreover, as the carrier rate of pathogenic *ABCA4* variants in the general population is relatively high [6, 27], carrier analysis is performed frequently to determine the risk for future offspring, and in these cases, it is important to know whether identified variants are pathogenic.

Databases, such as the *ABCA4*-Leiden Open (source) Variation Database (LOVD) [28] and ClinVar [29], provide a wealth of information for *ABCA4* variants including pathogenicity classifications. However, there is discordance between databases. The *ABCA4*-LOVD reports that up to 85% of the variants could be pathogenic, whereas in ClinVar, ~40% of the variants are reported to be likely pathogenic, which might be because many novel variants of uncertain significance are reported in ClinVar. Both the *ABCA4*-LOVD and ClinVar also allow for variable classifications as they are submitter-reported. Therefore, due to the variety of classification methods, it is difficult to truly assess and compare the pathogenicity of different variants.

A broadly used pathogenicity classification system is the ACMG/AMP classification as described by Richards et al. in 2015 [30]. This classification method incorporates information such as type of variant, *cis/trans* criteria, variant frequency, phenotype, functional studies, segregation, and *in silico* predictions. All information can be collected per variant and can easily be combined to a final pathogenicity classification with five tiers: benign, likely benign, variant of uncertain significance (VUS), likely pathogenic, or pathogenic. Since it is consistently used worldwide, it allows easy interpretation and comparison of pathogenicity levels.

In order to increase the knowledge on the pathogenicity of genetic variants in *ABCA4*, we collected all the data on *ABCA4*-AR cases that have been published up to 31 December 2020 and uploaded these data into the *ABCA4*-LOVD. Here, we adapted ACMG/AMP classifications incorporating ClinGen recommendations specifically for *ABCA4* and applied them to all 2,246 variants present in the *ABCA4*-LOVD.

## 2. Methods

We collected all papers published until 31 December 2020, which contain likely pathogenic *ABCA4* variants in individuals with retinopathy by searching the following search terms in PubMed:

(ABCA4[All Fields] OR ((“Stargardt disease”[All Fields] OR “Macula Lutea”[All Fields]) AND (“Genetics”[All Fields] OR “mutation”[All Fields] OR “Sequence Analysis”[All Fields] OR “gene panel”[TiAb]))) OR (“Retinal Dystrophies”[All Fields] AND (“mutation”[All Fields] OR “Sequence Analysis”[All Fields] AND “gene panel”[TiAb]))

Reported variants were collected per patient as well as additional available data such as gender, type of vision impairment, ethnicity, geographical origin, age at onset, phenotype at onset, segregation data, consanguinity status, and other remarks. The data were supplemented with data from 412 persons with *ABCA4*-AR from PreventionGenetics (a division of Exact Sciences). All data have been uploaded into the *ABCA4*-LOVD [28].

### 2.1. *ABCA4*-LOVD

All *ABCA4* variant data from the *ABCA4*-LOVD were downloaded on 5 April 2022.

### 2.2. Nomenclature

The annotation of all variants was done according to Human Genome Variation Society (HGVS) nomenclature guidelines where possible and is based on the GRCh37 hg19 genomic coordinates, gene location NM_000350.3. All variants were checked using the Batch Validator of the online VariantValidator tool [31]. Throughout the article, the c. notation of variants is used, supplemented with the p. notation, if available, when mentioned for the first time.

### 2.3. ACMG/AMP Classification

**Figure 1:**
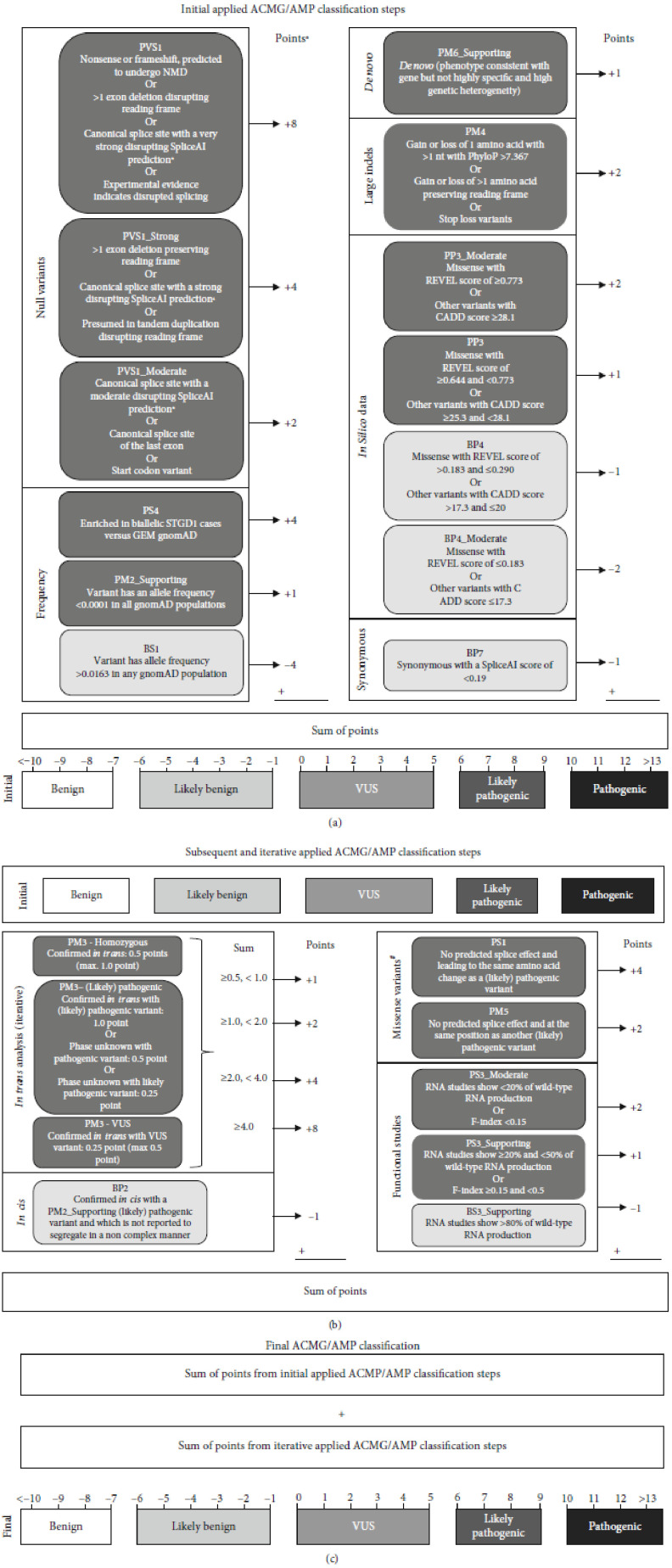
Applied ACMG/AMP classification steps incorporating ClinGen recommendations. (a) Initial steps taken to classify *ABCA4* variants from LOVD based on ACMG/AMP classification. For each step, a number of points are awarded and the sum of the points is used to determine the initial ACMG/AMP classification according to Tavtigian et al. [33]. GAM BAP gnomAD data are described in Cornelis et al. [27]. (b) Based on the initial ACMG/AMP classification, subsequent and iterative classification steps were executed. #The missense classification steps were not iterated to avoid circular reasoning. (c) The total sum of points lead to the final ACMG/AMP classification: benign (≤−7), likely benign (−6 to −1), VUS (0 to 5), likely pathogenic (6 to 9), and pathogenic (≥10).

All variants were classified according to ACMG/AMP variant classification guidelines described by Richards et al. [30], the updated recommendations from ClinGen [32], and the naturally scaled ACMG/AMP point system of Tavtigian et al. [33]. Our project group, consisting of experts on *ABCA4* genetics and ophthalmology, decided how best to apply the ACMG/AMP categories and ClinGen recommendations to *ABCA4* variants, which is summarized in Figure 1 and can be found in more detail in the Supplemental Materials & Methods (available here).

## 3. Results

### 3.1. Published *ABCA4*-AR data Cohort

After the removal of likely duplicates, the collection of data contained variants from 10,391 likely *ABCA4-*AR individuals, of which 3,411 were already reported in our 2017 study [34]. The cohort contained 6,240 likely biallelic cases and 4,151 monoallelic cases. Among these were 943 nonconsanguineous homozygous cases and 127 consanguineous homozygous cases. A total of 2,094 unique variants were identified in the cohort. All data were uploaded to the *ABCA4*-LOVD database [28].

### 3.2. ACMG/AMP Classification

**Figure 2:**
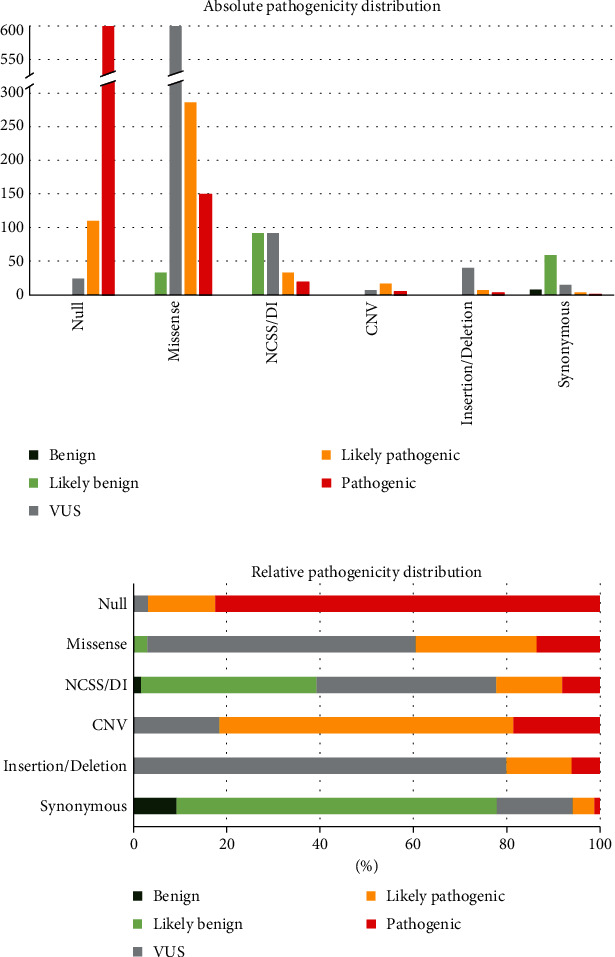
Distribution of ACMG/AMP classifications incorporating ClinGen recommendations among different types of mutations (null (loss of function), missense, noncanonical splice site (NCSS)/deep intronic (DI), copy number variation (CNV), (in-frame) insertion/deletion and synonymous). (a) Absolute occurrence of different pathogenicity classifications per mutation type. (b) Relative pathogenicity distribution among different mutation types.

ACMG/AMP classifications incorporating ClinGen recommendations were given to all variants from the published cohort as well as other *ABCA4* variants from the *ABCA4* LOVD (Table S1) and were annotated with the predicted pathogenicity severity according to Cornelis et al. [27] when available. Results of each ACMG/AMP classification step can be found in Tables S2-S10. After applying the noniterative classification steps, 1,276 variants could be categorized as benign, likely benign, likely pathogenic, or pathogenic. The iterative classification steps increased this number to 1,442 (Table 1 and Figure 2(a)). Of note, at the end of the analysis, 49 null variants reach “pathogenic” without the PVS1 criterium and >10% of variants associated with *ABCA4-*AR are loss of function confirming that the use of PVS1 flowchart is correct [35].

### 3.3. Null Variants

In total, 752 null variants were reported. Interestingly, 24 of these variants (3%) are still classified as VUS after the application of the ACMG/AMP classification system (Figure 2). Compared to other null alleles, the splice predictions for these 24 variants indicated that alternative in-frame splicing could occur or in-frame deletions were predicted. Furthermore, their occurrence in the dataset was too low to reach significance in the frequency analysis.

### 3.4. Missense Variants

A group of variants that are very difficult to interpret without an ACMG/AMP classification are missense variants. Here, we were able to classify 431 missense variants as (likely) pathogenic and 33 missense variants as likely benign. At the end of the analyses, 627 variants were classified as VUS (Figure 2).

### 3.5. Synonymous Variants

Of the 86 synonymous variants, five variants could be classified as (likely) pathogenic and 67 variants as (likely) benign (Figure 2).

### 3.6. Frequency Analysis

In the frequency analysis, 856 variants reached significant enrichment in the likely biallelic dataset compared to the earlier described [27] genetic ancestry matched (GAM) to biallelic affected persons (BAP) gnomAD control dataset after correction for multiple testing with the Benjamini–Hochberg method [36]. Interestingly, two variants, c.5603A>T and c.4253+43G>A ((p.[=,Ile1377Hisfs^∗^3])), were significantly enriched but had an odds ratio close to 1 (1.10 and 1.49, respectively) without having 1 in the confidence interval. This is a smaller odds ratio than 3, which is generally considered by Richards et al. [30] as the minimal odds ratio for variants with a modest Mendelian effect size. This effect is likely due to the reduced penetrance of these variants. Furthermore, 1,125 variants were not enriched in the likely biallelic dataset but had an allele frequency below 0.0001 in all gnomAD populations and therefore got “PM2_Supporting” evidence.

### 3.7. In Silico Analyses

SpliceAI scores were given to all variants < 50 nucleotides apart from indels. When possible, indels were given a CI-SpliceAI score. In total, 472 variants had a (CI-)SpliceAI score > 0.2 and received PP3 as minimal evidence. In parallel, REVEL and CADD scores were given to missense variants and other variants, respectively. In total, 1,141 variants received “PP3_Moderate” evidence, of which 658 variants were missense variants. On the lower end of the spectrum, 194 variants received “BP4_Moderate.” Interestingly, of those 194 variants, only 6 were missense variants, and 80 were other exonic variants (71 synonymous variants, 6 point deletions, and 3 in-frame duplications). Finally, 180 additional variants received PP3, and 48 variants received BP4. Of note, when comparing CADD and REVEL scores for missense variants based on the cut-offs as described by Pejaver et al. [37], it was noticed that only 7 variants received “BP4_Moderate” based on the cut-off of ≤0.183 for REVEL, while 124 variants would have received “BP4_Moderate” if the CADD score (cut-off of ≤17.3) would have been used (Figure S1).

### 3.8. Segregating Complex Alleles

The dataset contained a total of 65 unique alleles containing two or more variants (also known as complex alleles) for which segregation had been reported (Table S13). Of note, many individuals with multiple *ABCA4* variants were not reported to have undergone segregation analysis, resulting in a relatively low number of known complex alleles. The three most frequently reported complex alleles were c.[1622T>C;3113C>T] (p.[Leu541Pro;Ala1038Val]), c.[5461-10T>C;5603A>T] (p.[Thr1821Aspfs^∗^6,Thr1821Valfs^∗^13;Asn1868Ile]), and c.[2588G>C;5603A>T] (p.[Gly863Ala,Gly863del;Asn1868Ile]). Forty-three complex alleles were reported only once. Furthermore, twelve complex variants, including the three most frequent complex variants mentioned above, reached a likely pathogenic categorization based solely on the *in trans* analysis without correction for allele frequency. The nine other likely pathogenic complex allele variants were c.[302+68C>T;4539+2028C>T], c.[769-784C>T;5603A>T], c.[983A>T;3106G>A], c.[1715G>A;2588G>C], c.[3758C>T;5882G>A], c.[4222T>C;4918C>T], c.[4253+43G>A;6006-609T>A], c.[4469G>A;5603A>T], and c.[4926C>G;5044_5058del] (for protein notations, see Table S13). Of note, the pathogenicity scores of alleles with two variants do not have to reflect that both single variants have the same scores. Therefore, for reference, ACMG/AMP classifications of the single variants contained in the complex allele are given in Table S13 as well.

### 3.9. Frequent Pathogenic Variants

Two previously known pathogenic variants met the BS1 criterium—an allele frequency of >0.0163 in any gnomAD population—while also reaching a (likely) pathogenic classification. The c.5882G>A (p.(Gly1961Glu)) variant has an allele frequency of 0.023 in the gnomAD Ashkenazi Jewish population. The other variant is c.6320G>A (p.(Arg2107His)), which has an allele frequency of 0.021 in the gnomAD African population. For each gnomAD population, the three most frequent (likely) pathogenic variants are reported in Table S11. Interestingly, three relatively less well-known variants, c.2791G>A (p.(Val931Met)), c.2971G>C (p.(Gly991Arg)), and c.6320G>A, have a very high frequency (0.004-0.021) in the gnomAD African population, while they are less frequent in other populations. Similarly, in the Latino/Admixed American population, variant c.872C>T (p.(Pro291Leu)) with an allele frequency of 0.0038 was found to be likely pathogenic. Given their high allele frequencies, it will be of interest to investigate if they show reduced penetrance, which might not be unexpected as all of these variants were predicted to be mild [27, 38]. Previously reported frequent pathogenic variants and their updated ACMG/AMP classification can be found in Table S12. Interestingly, the variant c.2588G>C, earlier described as “North European,” has a high frequency in the gnomAD South Asian population as well.

## 4. Discussion

In this study, we assigned ACMG/AMP classifications other than VUS to 1,442 of 2,246 *ABCA4* variants based on the point system of Tavtigian et al. [33] and ClinGen recommendations [32], which is more than twice as much as our 2017 classification [34]. Compared to the 2017 classification, 1,419 new variants were analyzed. From the previously analyzed variants, 210 variants got a more severe pathogenicity score, and 142 got a more benign pathogenicity score. In total, 93 variants are now classified as VUS, while the 2017 study assigned a different classification to them. These differences are likely the result of an increase in available knowledge, such as information from new functional studies and improved prediction software, as well as a more gene-specific approach of applying the ACMG/AMP rules. In total, 1,248 variants were classified as either likely pathogenic or pathogenic, 804 variants were classified as VUS, and 194 variants were classified as either likely benign or benign. Furthermore, Table S1 provides a framework that can easily be adjusted to improve the AMP/ACMG classification of *ABCA4* variants when additional information becomes available. This will be of important value to clinical geneticists, individuals affected by *ABCA4*-AR, their family members, and ongoing clinical trials for gene-specific therapies.

### 4.1. The Limitation of ACMG/AMP Classification for *ABCA4* Variants

The ACMG/AMP classification is designed in a way that likely pathogenic and likely benign variants are classified with ≥90% certainty. Although this is usually interpreted as a very reliable classification, it should be mentioned that when a large group of variants is classified, some variants will receive an incorrect classification. Furthermore, it is important to mention that the dichotomous pathogenicity framework that the ACMG/AMP classification system is based on currently categorizes a variant as pathogenic if it can cause disease, even if it does not always cause disease *in trans* with another pathogenic variant, such as for the reduced penetrant variants that have been reported in *ABCA4* [15]. Recently, multiple studies have shown that using the Mendelian model of traits being either recessive or dominant limits the understanding of the role genetic variants have in disease mechanisms that show a gradual or varying effect [39, 40]. It has, therefore, been suggested to expand the ACMG/AMP classification to a seven-tier system including “predisposing” and “likely predisposing” as additional classifications [39]. The term “predisposing” may indeed be a better classification for variants with reduced penetrance than the dichotomous term “pathogenic.” However, for most variants, it will be difficult to determine whether they show reduced penetrance, leading to a “Predisposing” classification, or not, leading to a “pathogenic” classification. Here, we annotated variants that have been reported to show reduced penetrance (Table S1). Reduced penetrance is reflected in both discordance in families with *ABCA4*-AR as well as in a low odds ratio in enrichment studies, e.g., below 28.1 in this study (Table S4). It may, therefore, be advisable to be cautious for pathogenic and likely pathogenic variants with an odds ratio < 28.1 in particular, as these might be variants with reduced penetrance.

Interestingly, the well-known variant c.5603A>T, which shows a very low penetrance when *in trans* with a severe or null allele (approximately 5%) [14] is classified as a VUS. This variant was long believed to be benign as it was found to cooccur with the less frequent variant c.2588G>C which was thought to be pathogenic [41]. However, in 2017, Zernant et al. identified that c.5603A>T is disease causing, and c.2588G>C without c.5603A>T might not be pathogenic [26]. It was clear that c.5603A>T was underreported in the data from before 2017 because of this, since c.2588G>C has often been reported without c.5603A>T in the literature. This means that the pathogenicity of c.2588G>C is likely overclassified here, while that of c.5603A>T is likely underclassified. Similarly, the mild variant c.4253+43G>A, which shows a splice defect *in vitro* [16] and shows reduced penetrance [42], is classified as VUS. A possible explanation for this is that deep intronic variants—including the ones reported by Braun et al. in 2013, although to a lesser extent [43]—are underreported in literature, since targeted *ABCA4* exon sequencing or WES were the norm for a long time and additionally because their interpretation can be challenging. Therefore, they are less likely to reach significant enrichment in the dataset in step PS4 and may be excluded from the PM3 criterium *in trans* classification when they are not reported. Of note, new and more affordable techniques are now increasing the number of identified deep intronic variants [16, 44, 45], which will likely improve the knowledge on their pathogenicity. However, since those techniques are not yet available everywhere, it will be challenging to identify the pathogenicity of deep intronic variants in all populations.

This illustrates the difficulty of recognizing frequent mild pathogenic variants that show reduced penetrance. Therefore, variants with an odds ratio between 1 and 3 should particularly be treated with caution, although reduced penetrance has been predicted for variants with an odds ratio up to 28.1 [15]. Larger studies are necessary to identify whether these variants show reduced penetrance or that instead, they may be in linkage disequilibrium with an unknown pathogenic variant. The distinction between these may in part be predicted by the variants identified *in trans*; if the majority of those are severe, the variant or a variant in linkage disequilibrium with it is likely mild and might show reduced penetrance. However, if the variants *in trans* are not consistently severe, then the variant is likely in linkage disequilibrium with an unidentified (moderately) severe variant.

Modifiers seem to play a role in *ABCA4*-AR and may explain the occurrence of variants with reduced penetrance. A sex imbalance has been reported for individuals having mild likely reduced penetrant variants [15], and common *PRPH2* variants and rare *ROM1* variants have been reported to act as modifiers of *ABCA4*-AR [46]. In three Dutch families with biallelic sibling pairs carrying c.5603A>T *in trans* with another severe *ABCA4* variant, not all siblings were affected by *ABCA4*-AR [14]. Kjellström and Andréasson may also have found two unaffected male individuals over 50 that had both c.5603A>T and a severe variant [47]. Modifiers may similarly explain the reported high variety in the disease course of *ABCA4*-AR [48]. Furthermore, modifiers might also aggravate the *ABCA4-*AR phenotype of individuals; Leber's congenital amaurosis, causing severe vision loss in the first year of life, is usually not associated with variants in *ABCA4*, but Panneman et al. identified probands in which two *ABCA4* null alleles are hypothesized to cause Leber's congenital amaurosis [49] (Panneman, Koenekoop, Cremers, unpublished data).

Another important point to raise for recessive disease is that variants leading to a protein with reduced but not abolished expression and/or function may not always be disease causing, depending on the variant *in trans*. For example, if a variant reduces protein expression to 45% compared to WT, then its occurrence next to a null variant is likely disease causing. A homozygous occurrence of this variant, however, will lead to expression only just below that of an individual with a null allele in addition to a WT allele. This may cause a situation similar to a combination of a hypomorphic variant next to a null allele, where modifiers may determine whether an individual will be affected or not. In other words, considering that all *ABCA4* variants are on a spectrum based on residual protein function and resulting cellular dysfunction, it is likely that the combined severity of *ABCA4* variants and modifiers together determine disease penetrance and severity.

Furthermore, several studies indicate that pathogenic variants in other genes can be responsible for *ABCA4-*AR even when one likely pathogenic *ABCA4* variant is present in the patient. Disease-causing variants can sometimes be found in genes like *PRPH2* and *PROM1,* but also in less common genes associated with *ABCA4*-like diseases like *BEST1*, *CDHR1*, *CERKL*, *CNGA3*, *CRX*, *ROM1*, and *RPE65* [19, 20, 50].

### 4.2. Study Limitations

Apart from the aforementioned limitation that the ACMG/AMP guidelines have when considering variants with a gradual versus a dichotomous pathogenicity effect, there are a few more limitations to this study. The first one is associated with PS4, the allele frequency analysis. First, the GAM BAP control dataset used is based on the reported ethnicity, which may not correspond with the gnomAD population that individuals were matched with, since ethnicity is a social construct and gnomAD populations are for a big part based on principal component analyses [51], and it is unknown to what extent those overlap. Second, for those patients without reported ethnicity, the GAM BAP gnomAD has incorporated estimated ethnicity based on population statistics. However, there may be a bias in the ethnicity of patients who are able to either afford healthcare, who have the option to take part in a study, or who feel safe to take part in a study since, for example, historic transgressions have been made against Black research participants [52]. Finally, as labs that study *ABCA4*-AR and report individuals with *ABCA4-*AR are unequally distributed over the world, a bias in the genetics of subpopulations of those regions could occur. Therefore, it is likely that population stratification will have affected the results of the allele frequency test. In order to improve healthcare for everyone, it is, therefore, important that rare genetic variants in individuals with underreported genetic ancestry in literature are studied more to improve the knowledge on all genetic variants, and that variants with a high-frequency difference between populations are investigated more closely to study their effect since differences in identified variants and numbers of *ABCA4-*AR cases between different ethnicities have been reported [53]. Currently, both genetic and disease data of white individuals with *ABCA4-*AR are overrepresented, creating an imbalance in understanding of the genetic cause of *ABCA4*-AR and treatment options between these individuals and individuals of color with *ABCA4*-AR.

Moreover, based on the final classification, 298 total variants from the biallelic dataset (2.3%) are likely benign or benign. This means that up to 298 cases from this dataset are not actually known biallelic, which might indicate that those cases are not actually *ABCA4*-AR cases, which could create a bias in the enrichment analysis for the variants present *in trans*.

Furthermore, the ACMG/AMP guidelines and recommendations warn for the use of functional studies. We indeed encountered that a variant, c.4539+2028C>T (p.[=,Arg1514Leufs^∗^36]), which likely is a pathogenic variant based on genotype-phenotype correlations [43], shows a higher percentage of WT RNA in patient-derived retinal-like cells than expected [54]. We decided to remove this data point from the dataset as an outlier in the classification based on functional studies in step BS3. In addition, other variants, e.g., c.4539+2001G>A (p.[=,Arg1514Leufs^∗^36]) and c.1937+435C>G (p.[=,Ser646Serfs^∗^25]), show a relatively high percentage of WT RNA production, 75% and 55%, respectively, in patient-derived retinal-like cells [54] and midigene assays, respectively [16], while genotype-phenotype correlations show that these variants are likely pathogenic [43, 55]. This indicates that other variants could show a similar pattern, meaning that intronic variants causing a relatively high amount of WT RNA may nevertheless be pathogenic. Therefore, results from midigene assays and patient-derived retinal-like cells should be interpreted with caution. However, since most variant results seem to correlate with their pathogenicity, BS3_Supporting was deemed to be of proper evidence strength.

In addition, in the use of *in silico* predictions, the CADD score was used for nonmissense variants. However, the applied cut-offs were based on the study of Pejaver et al. [37] in which only missense variants were studied. Furthermore, when comparing REVEL and CADD scores for *ABCA4* missense variants, CADD scores seem to lead to a more benign category (Figure S1). Only one variant that got PP3_Moderate because of its REVEL score would have gotten BP4_Moderate if CADD would have been used. However, since REVEL is specialized in scoring missense variants and as it outperformed CADD in two studies [37, 56], the REVEL scores were considered to be more trustworthy. Therefore, we decided to increase the range of CADD scores leading to no *in silico*evidence score from 22.7-25.3 to 20-25.3 to avoid incorrectly classifying in-frame insertions/deletions, noncanonical splice variants, and synonymous variants as benign since 20 is often used as a cut-off between a benign and a pathogenic indication. In the final categorization, this led to four variants being categorized as likely pathogenic or pathogenic instead of a lower category.

Finally, it should be mentioned that variant classification is, and should be, dynamic. Since the initial ACMG/AMP guidelines were published in 2015 [30], new insights have led to many recommendations to improve the classification system [32, 33, 37, 39]. With the increasing knowledge on genetic disease and improving strategies to understand variant effects, it is, therefore, important to regularly incorporate evolving variant classification strategies.

### 4.3. Future Scope

Finally, the *ABCA4* variant dataset analyzed here mostly stems from 421 peer-reviewed publications as well as data from PreventionGenetics (a division of Exact Sciences). In the future, it would be very valuable to include variant data collected in all academic and nonacademic diagnostic centers worldwide. This will be challenging as privacy rules may prevent data sharing and differences between rules in different countries likely create a bias in the data. Furthermore, data currently existing in different online databases may show overlap and are not all curated.

With the advent of novel therapies, it is essential to have an accurate genetic diagnosis, which emphasizes the importance of the classification of variants and proper guidelines. *ABCA4* variant classification is challenging due to the higher mutation frequency, presence of complex alleles, and hypomorphic variants with reduced penetrance. The adapted ACMG/AMP classifications provided in this study, in combination with the earlier established severity assessments for *ABCA4* variants, will facilitate the interpretation of diagnostic results for *ABCA4*-AR, the most common recessive retinal disease.

## Figures and Tables

**Table 1:** Overview of the number of *ABCA4* variants from LOVD per ACMG/AMP categorization.

ACMG/AMP category	Initial (before iteration steps)	Final
Benign	8 (0.4%)	12 (0.5%)
Likely benign	191 (8.5%)	182 (8.1%)
Variant of uncertain significance	971 (43.2%)	804 (35.8%)
Likely pathogenic	498 (22.2%)	452 (20.1%)
Pathogenic	579 (25.8%)	796 (35.4%)

## Data Availability

The collected data from affected individuals from literature used to support the findings of this study have been deposited in the ABCA4-LOVD: http://www.lovd.nl/ABCA4.

## Supplementary Materials

Supplementary Materials A file containing supplemental material pertinent to this manuscript is available. This supplemental information consists of 13 tables, 2 figures, and detailed methods, as follows: Table S1: all variants with ACMG/AMP classifications (column H) and severity subclassifications from [27] (column I). ACMG/AMP classifications are based on the point system as described by Tavtigian et al. [33]. In short, supporting, moderate, strong, and very strong evidence is combined into a score where each type of evidence gives a score of 1, 2, 4, or 8, respectively, where pathogenic evidence gives a positive score and benign evidence gives a negative score. The resulting total score per variant results in a benign (<-6), likely benign (-1–-6), VUS (0–5), likely pathogenic (6–9), or pathogenic (>9) classification. Table S2: ACMG/AMP classification step PVS1 Null variants. Table S3: ACMG/AMP classification step PM6 *de novo* variants. Table S4: ACMG/AMP classification step PS4 variant frequency and use of control populations. Table S5: ACMG/AMP classification step PM4 protein length changes due to in-frame deletions/insertions and stop losses. Table S6: ACMG/AMP classification steps PP3 and BP4 computational (*in silico*) data. Table S7: ACMG/AMP classification step BP7 synonymous variants. Table S8: ACMG/AMP classification steps BS1 and PM2 variant frequency and use of control populations. Table S9: ACMG/AMP classification steps PS1 and PM5 Same amino acid change and novel missense at the same position. Table S10: ACMG/AMP classification steps PS3 and BS3 functional studies. Table S11: three most frequent (likely) pathogenic variants per gnomAD population. Table S12: previously reported frequent pathogenic variants based on literature. Table S13: published segregating complex alleles. Figure S1: *in silico* comparison of CADD and REVEL for missense variants in *ABCA4. In silico* comparison of *ABCA4* missense variants. CADD PHRED values are plotted against REVEL values. Cut-off values between “BP4_Moderate” and “BP4,” and between “PP3” and “PP3_Moderate” are shown in green and red, respectively, vertically for CADD scores and horizontally for REVEL scores. Overall, it can be observed that the variants reach a higher category for REVEL than for CADD. Figure S2: correlation between variant F-indexes and age of onset in patients. Correlations between *ABCA4* variants' F-index and the corresponding log 10 and square root transformed age at onset for homozygous configuration (A) and compound heterozygous configuration with a severe variant (B).
