# Corrigendum to “Compendium of Clinical Variant Classification for 2,246 Unique *ABCA4* Variants to Clarify Variant Pathogenicity in Stargardt Disease Using a Modified ACMG/AMP Framework”

**DOI:** 10.1155/humu/9815325

**Published:** 2025-09-12

S. S. Cornelis, M. Bauwens, L. Haer-Wigman, et al., “Compendium of Clinical Variant Classification for 2,246 Unique *ABCA4* Variants to Clarify Variant Pathogenicity in Stargardt Disease Using a Modified ACMG/AMP Framework,” *Human Mutation* 2023 (2023): 6815504, https://doi.org/10.1155/2023/6815504.

In the article titled “Compendium of Clinical Variant Classification for 2,246 Unique *ABCA4* Variants to Clarify Variant Pathogenicity in Stargardt Disease Using a Modified ACMG/AMP Framework,” there was an error in Figure 1 artwork and caption. The corrected Figure 1 artwork and caption are shown below:

We apologize for this error.

## Figures and Tables

**Figure 1:**
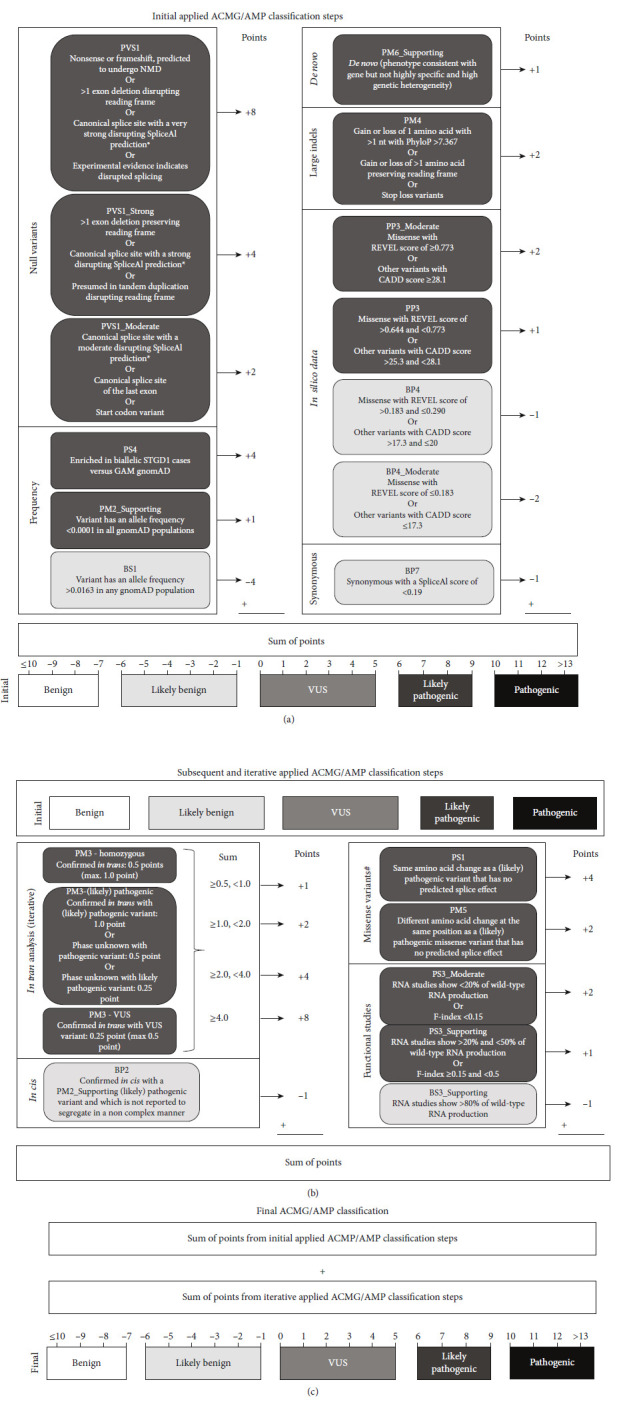
Applied ACMG/AMP classification steps incorporating ClinGen recommendations. (a) Initial steps taken to classify *ABCA4* variants from LOVD based on ACMG/AMP classification. For each step, a number of points are awarded and the sum of the points is used to determine the initial ACMG/AMP classification according to Tavtigian et al. [33]. GAM BAP gnomAD data are described in Cornelis et al. [27]. ∗See “PVS1, PVS1_Strong, and PVS1_Moderate based on splice predictions” in the Supporting Information for further explanation. (b) Based on the initial ACMG/AMP classification, subsequent and iterative classification steps were executed. ^#^The missense classification steps were not iterated to avoid circular reasoning. Furthermore, the absence of splice prediction changes refers to the known (likely) pathogenic variant, not to the variant of interest. (c) The total sum of points leads to the final ACMG/AMP classification: benign (≤ −7), likely benign (−6 to −1), VUS (0–5), likely pathogenic (6–9), and pathogenic (≥ 10).

