# Supplementary material for: Compendium of Clinical Variant Classification for 2,246 Unique ABCA4 Variants to Clarify Variant Pathogenicity in Stargardt Disease Using a Modified ACMG/AMP Framework
Source: Hum Mutat. 2023 Dec 26;2023:6815504. doi: 10.1155/2023/6815504 (PMC11918811; doi:10.1155/2023/6815504)
Supplement: Supplementary Materials — A file containing supplemental material pertinent to this manuscript is available. This supplemental information consists of 13 tables, 2 figures, and detailed methods, as follows: Table S1: all variants with ACMG/AMP classifications (column H) and severity subclassifications from [27] (column I). ACMG/AMP classifications are based on the point system as described by Tavtigian et al. [33]. In short, supporting, moderate, strong, and very strong evidence is combined into a score where each type of evidence gives a score of 1, 2, 4, or 8, respectively, where pathogenic evidence gives a positive score and benign evidence gives a negative score. The resulting total score per variant results in a benign (<-6), likely benign (-1–-6), VUS (0–5), likely pathogenic (6–9), or pathogenic (>9) classification. Table S2: ACMG/AMP classification step PVS1 Null variants. Table S3: ACMG/AMP classification step PM6 de novo variants. Table S4: ACMG/AMP classification step PS4 variant frequency and use of control populations. Table S5: ACMG/AMP classification step PM4 protein length changes due to in-frame deletions/insertions and stop losses. Table S6: ACMG/AMP classification steps PP3 and BP4 computational (in silico) data. Table S7: ACMG/AMP classification step BP7 synonymous variants. Table S8: ACMG/AMP classification steps BS1 and PM2 variant frequency and use of control populations. Table S9: ACMG/AMP classification steps PS1 and PM5 Same amino acid change and novel missense at the same position. Table S10: ACMG/AMP classification steps PS3 and BS3 functional studies. Table S11: three most frequent (likely) pathogenic variants per gnomAD population. Table S12: previously reported frequent pathogenic variants based on literature. Table S13: published segregating complex alleles. Figure S1: in silico comparison of CADD and REVEL for missense variants in ABCA4. In silico comparison of ABCA4 missense variants. CADD PHRED values are plotted against REVEL values. Cut-off values between [file 6815504.f1.zip › Supplemental Methods.docx]

**Supplemental Methods**

ACMG/AMP classifications steps give evidence for variants being pathogenic (P) or benign (B) with different strengths of evidence (Very Strong (VS), Strong (S), Moderate (M) or Supporting (P)) as described in Richards et al. 2015 [1]. These letters are combined and given a number to describe different types of evidence (e.g. PVS1 stands for ‘Pathogenic’ evidence that is ‘Very Strong’, type ‘1’). Since 2015, multiple recommendations have been made to increase or decrease the evidence strengths of different types. In these recommendations underscores were added to the original name of the original evidence type, followed by the new recommended strength [2] (e.g. PVS1 was subdivided into PVS1, PVS1_Strong and PVS1_Moderate, depending on predictions of canonical splice site variant effects). Therefore, the evidence strength mentioned after the underscore is the evidence strength that is applied. In this article, we made use of point system introduced by Tavtigian et al. in 2020 [3]. In short, a variant gets positive points for pathogenic evidence and negative points for benign evidence. Very Strong evidence gives or subtracts 8 points, Strong 4 points, Moderate 2 points and Supporting 1 point. The total amount of points granted to a variant will lead to the final pathogenicity category as shown in Figure 1. For each ACMG/AMP classification step the specific application to *ABCA4* data as applied in this study is described below.

Excluded evidence types

The classification steps "PM1 Mutational hot spot and/or critical and well-established functional domain”, "PP2 BP1 Variant spectrum", "PP4 Using phenotype to support variant claims" as well as the criterium "BS2 Use of control populations" were not applied in this study since these steps are not applicable for *ABCA4*. “PP1 BS4 Segregation analysis” was not used in this study because these data were not consistently available.

Iterated evidence types

The steps "PS1 Same amino acid change", "PM3 BP2 Cis/trans testing", "PM5 Novel missense at the same position" and "PS3 BS3 Functional studies" were executed at the end and repeated as described to increase the number of variant classifications in the categories pathogenic, likely pathogenic, likely benign and benign.

*PVS1 PVS1_Strong PVS1_Moderate Null Variants*

Null variants were categorized as described by Abou Tayoun et al. [4]with some exceptions as described below.

For canonical splice site variants, it is indicated to estimate the impact of the variant on the open reading frame. Where possible, this was based on experimental data. If those data were not available the SpliceAI prediction software was used, as it was proven to be the most accurate for *splicing* predictions of non-canonical splice site variants in *ABCA4* [5] and as it is able to incorporate a wider context of the gene compared to most other splice prediction tools [6].

*PVS1 based on experimental data of splice variants*

The PVS1 classification was allocated to all canonical splice site variants that were in the direct vicinity (at a maximum of ten nucleotides away) of non-canonical splice site variants that were experimentally proven to cause splicing defects that lead to the disruption of the reading frame in midigene assays [7].This is based on the assumption that if a non-canonical splice site change disrupts the splice site completely, a canonical splice site change will most likely also fully disrupt the splice site.

*PVS1, PVS1_Strong and PVS1_Moderate based on splice predictions*

Canonical splice sites for which no such experimental data were available, were annotated based on SpliceAI predictions. Based on these predictions, the possibility of in-frame and out-of-frame exon skipping as well as alternative splicing was considered. In short, SpliceAI scores were used to predict the effect of the variants on new splice site creation as well as canonical splice site loss. Small indels, which cannot get a score in SpliceAI, were annotated with CI-SpliceAI scores [8], computed with the online CI-SpliceAI tool [9]. A delta score cut-off of ≥0.2 was used to estimate a true predicted effect, which is slightly more conservative than the cut-off calculated by Riepe et al [5]. Predicted exon skipping was only considered to be true if both canonical splice site variants of the exon were predicted to be lost. Predicted exon elongation or shortening was considered to be true only if both a splice site was lost and a new splice site was predicted. The following evidence categories were assigned:

- PVS1: large in-frame deletions (>50% of the gene) that included the loss of the start codon and canonical splice site variants that were predicted to lead to out-of-frame exon skipping, out-of-frame exon elongation, out-of-frame exon shortening, or would lead to elongation creating a stop codon.
- PVS1_Strong: Variants that were predicted to lead to the loss of only one splice site without having a new splice site predicted, variants where two splice sites were lost but only one alternative splice site was predicted and variants predicted to lead to in-frame exon skipping based on the assumption that all exons in *ABCA4* are critical to protein function.
- PVS1_Moderate: Variants that were predicted to lead to an in-frame exon elongation/shortening without creating a new stop codon and canonical variants at the acceptor splice site of the last exon based on the prediction of an introduction of 26 random amino acids from the 3’ untranslated region.

*PS4 PM2_Supporting BS1 Variant Frequency and Use of Control Populations*

Due to known reduced penetrance in *ABCA4*, BA1, which is the stand alone criterium of benign impact for variants with an allele frequency >0.05, was not used in this study and the frequent variant c.5603A>T (p.(Asn1868Ile)) was submitted to the Exception List Nomination Form for a BA1 modification as suggested elsewhere [11]. For BS1 a cut-off allele frequency of 0.0163 was used based on the Whiffin/Ware calculator [12] with the conservative settings of a prevalence of 1:7,500, allelic and genetic heterogeneity of 1 and penetrance of 50%. Variants that met this cut-off in any of the gnomAD populations were assigned the BS1 category. The PM2 criterium indicates that if a variant is absent or extremely rare in a control population, there is evidence for this variant to be pathogenic. The evidence is decreased to ‘PM2_Supporting’ according to the SVI Recommendation for Absence/Rarity (PM2) - Version 1.0 [13]. ‘PM2_Supporting’ was applied to variants with an allele frequency <0.0001 in each of the gnomAD populations, based on the observation that ~95% of the variants categorized as (likely) pathogenic in Cornelis et al., 2017 [14, 15] have an allele frequency of <0.0001 and no variants categorized as (likely) benign had an allele frequency <0.0001. ‘PM2_Supporting’ was only assigned if PS4 was not already assigned.

Selecting data for the frequency analysis

For the allele frequency analysis, we included individuals with two or more variants that were not homozygous consanguineous cases. Before the analysis, we compared the entries of affected individuals to one another to identify possible duplicates. Cases having the same ≥2 variants, that showed to have a different gender, different age at onset or different ethnicity, were determined to be unique individuals. Whenever the remaining possible duplicates were published by at least one overlapping author, entries were removed from the dataset. Thereafter, data from individuals were excluded from the dataset when no segregation analysis had been performed and exactly two variants were identified that were found as part of a complex allele at least twice in the dataset (Table S13) or if it that was likely to be the c.[769-784C>T;5882G>A] (p.[=,Leu257Aspfs*3; Gly1961Glu]) allele [16]. Mono-allelic cases were excluded for the two reasons that they might not be *ABCA4*-retinopathy cases and that they likely contain duplicate entries. Finally, 127 alleles from 127 homozygous consanguineous cases were added to the data. Only one allele per individual was included as both alleles most likely have the same origin and therefore are not independent alleles. To avoid bias, only independent datapoints (alleles in this analysis) can be used in the statistical analysis.

Frequency analysis

The variant frequency of the biallelic *ABCA4*-retinopathy individuals was compared to the earlier published biallelic affected person (BAP) based genetic ancestry matched (GAM) gnomAD control group [17] and was tested with a one-tailed Fisher’s Exact test. P-values were corrected with the False Detection Rate from Benjamini and Hochberg 1995 [18]. In short, the BAP GAM gnomAD control group is a control group that endeavors to match the genetic ancestry of published biallelic *ABCA4­*-retinopathy affected individuals based on reported genetic ancestry and estimated genetic ancestry. This control dataset was based on gnomAD version 2.1.1. To determine the allele count in the BAP GAM gnomAD control group, we multiplied the BAP GAM gnomAD allele frequency with the estimated allele number of the BAP GAM gnomAD control group for that location based on the limiting population size as described in Cornelis et al., 2022. For variant locations absent in gnomAD we used the median Allele Number of the BAP GAM gnomAD dataset of either exonic and near exonic variants from biallelic *ABCA4*-retinopathy individuals, near exonic being <100 nucleotides away from the exon, or intronic to estimate the allele number on the genomic position of the variant. For structural variants, insertions and deletions >49 base pairs, we used the median of structural variants in gnomAD, since population data were not downloadable.

*PM6_Supporting De novo variants*

Reported *de novo* variants (n=3) were classified with the PM6 criterium according to the recommendation [svi_proposal_for_de_novo_criteria_v1_1.pdf (clinicalgenome.org)](https://clinicalgenome.org/site/assets/files/3461/svi_proposal_for_de_novo_criteria_v1_1.pdf). Since it was impossible to check the identity of the parents of affected individuals with *de novo* variants, PM6 was applied instead of PS2. According to the recommendations, 0.5 points were attributed to each *de novo* variant with unconfirmed parental relationships, based on the phenotype consistency category “Phenotype is consistent with the gene but not highly specific”, which led to PM6_Supporting for all *de novo* variants in this dataset. Of note, two reported *de novo* variants, c.3106G>A (p.(Glu1036Lys)) and c.4217A>G (p.(His1406Arg)), were excluded. These were reported in the same individual, which seems unlikely since the variant c.983A>T (p.(Glu328Val)) was found in this individual as well, which is known to occur as a complex allele with c.3106G>A, i.e. c.[983A>T;3106G>A]. This raises the possibility of a sample error [10].

*PM4 Large In-Frame Deletions Insertions In Conserved Areas*

The PM4 category was assigned to variants leading to a stop loss, variants affecting more than one amino acid or variants affecting one amino acid in which two nucleotides had a PhyloP (derived from the UCSC Tabel Browser: Comparative Genomics; name="100 Vert. Cons" description="100 vertebrates Basewise Conservation by PhyloP") [19, 20] of 7.367 or higher, which was based on the PhyloP Supporting *in silico* strength from Pejaver et al., 2022 [21].

*PP3_Moderate PP3 BP4 BP4_Moderate Computational In Silico Data*

Although Richards et al. suggest to only assign PP3 when all used *in silico* predictions indicate pathogenicity [1], it was decided to perform the *in silico* analysis for splicing and other predictions in parallel. The highest resulting pathogenic criterium was assigned. SpliceAI was used to predict splicing effects. A maximal distance of 2,000 nucleotides between variant and effect was used and only the strongest prediction was considered. For insertion deletion variants, which cannot get a score from SpliceAI, the online tool CI-SpliceAI was used [8]. A cut-off of ≥0.2 was used to determine whether a prediction was considered a likely true effect and lead to assignment of PP3. Finally, PP3 was not assigned to canonical splice site variants, since those were already assigned a PVS1 criterium based on SpliceAI predictions unless the predictions were overruled by experimental data as described above.

In parallel, REVEL was used for missense variants [22] and CADD for other variants affecting up to 50 bp [23, 24] as indicated in the table below. These cut-offs are based on Pejaver et al., 2022 [21] with the exception of the BP4 category of CADD, for which an upper cut-off value of 20 was applied, a widely used cut-off for pathogenicity. Variants >50 bp did not undergo an *in silico* prediction (Supplemental Methods Table 1).

**Supplemental Methods Table 1 Cut-off values for *in silico* data**

|  | **REVEL (missense)** | **CADD (non-missense, <50bp)** |
| --- | --- | --- |
| **PP3_Moderate** | ≥0.773 | ≥28.1 |
| **PP3** | ≥0.644 and <0.773 | ≥25.3 and <28.1 |
| **BP4** | >0.183 and ≤0.290 | >17.3 and ≤20 |
| **BP4_Moderate** | ≤0.183 | ≤17.3 |

# For *in silico* programs requiring a vcf input file, [GitHub - HGVS variant name parsing and generation](https://github.com/counsyl/hgvs) was used (downloaded in August 2021) [25]. Duplication variants were manually adjusted when necessary.

*BP7 Synonymous Variants*

Synonymous variants that are unlikely to create splicing defects were assigned BP7. Many *ABCA4* variants located at the outer sides of exons are known to create splice defects. Therefore, all synonymous variants located at the first three or last three nucleotides of an exon were excluded from the BP7 criterium. Furthermore, synonymous variants with a SpliceAI prediction of 0.19 or higher were also excluded from being assigned BP7 based on Riepe et al [5].

**Iterated steps**

The following evidence categories depend on the classification of other variants and were therefore executed at the end, some with several iterations.

*PM3_Supporting PM3 PM3_Strong PM3_VeryStrong BP2 Cis/trans* *Testing*

The PM3 category was performed first according to the SVI Recommendation for in trans Criterion (PM3) - Version 1.0. In short, variants occurring in likely biallelic individuals were assessed. Depending on the pathogenicity of the variant a variant of interest occurs with, the variant of interest gets a score between 0.0 and 1.0, depending on phasing data. For example, if a variant of interest is confirmed to be *in trans* with a known pathogenic null variant, then the variant of interest gets 1.0 point for this occurrence. To avoid wrongly assigning a pathogenic criterium to a variant of interest occurring *in* *trans* with a pathogenic variant by chance due to the high frequency of the latter, the variant of interest did not get any points assigned to it if the pathogenic variant *in trans* did not meet the PM2 criterium - occurring with an allele frequency less than 0.0001. For variants that occurred with an allele frequency of >0.0001 themselves we applied the following correction: the number of points assigned to those variants was multiplied with 0.0001 divided by the allele frequency of the variant. The complex alleles c.[1622T>C;3113C>T] (p.[Leu541Pro;Ala1038Val]) and c.[5461-10T>C;5603A>T] were added to the list of pathogenic variants since they are well-known and frequently occurring penetrant pathogenic alleles.

The BP2 criterium was subsequently applied to variants that occurred with a rare (PM2) (likely) pathogenic variant *in* *cis* and that was not reported to occur as a single variant based on phasing data.

*PS1 Same amino acid change*

Variants that caused the same amino acid change as a known likely pathogenic or pathogenic variant that is not known to cause a splicing effect were assigned PS1.

*PM5 Novel missense at the same position*

Variants that caused a different amino acid change at the same position as another missense change that was assigned likely pathogenic or pathogenic that is not known to cause a splicing effect were assigned PM5.

*PS3_Supporting PS3_Moderate BS3_Supporting Functional Studies*

The PS3 and BS3 criteria were assigned based on the recommendations of Brnich et al. [26]. It was decided that the disease mechanism for STGD1 is understood enough and that midigene assays as well as protein assays model the disease well enough for use of this criterium. These assays contained both wild-type (WT) cDNA constructs and proteins, and cDNA constructs and proteins having known pathogenic variants. Since the assays have been broadly accepted historically, the use of multiple replicates was not necessary.

Midigene assay data

First, functional data from midigene assays were assessed according to Brnich et al. Figure 1: looking at both the highest amount of residual WT RNA of non-missense likely pathogenic and pathogenic classified variants and the lowest amount of WT RNA of likely benign and benign classified variants. However, since there was an overlap between (likely) benign and (likely) pathogenic variants in terms of produced WT RNA in the range of 61-76% of WT RNA, we decided on assigning evidence types as mentioned in Supplemental Methods Table 2. The ‘PS3_Moderate’ category is higher than advised by Brnich et al., but it was decided that for these functional assays <20% WT RNA is strong enough evidence to reach Moderate. The variant c.4539+2028C>T, which shows 85% WT RNA expression in photoreceptor progenitor cells [27], was excluded from this list since genotype-phenotype correlations indicate that it is pathogenic. As observed for a few variants that result in a ‘retina-specific’ or ‘retina-enhanced’ splice defect, photoreceptor progenitor cells and retinal organoids do not always show the complete splice defect that is present in the proband’s retinae [27, 28].

F-index data

Known (likely) pathogenic missense variants that have an F-index as published by Curtis et al., were used to determine an F-index cut-off value for assigning PS3 to missense variants [29]. It was attempted to assess the data as indicated by Brnich et al., but similarly to the midigene assay data an overlap is observed between pathogenic variants and the WT F-index value. However, since the log10 and square root transformed average age at onset of individuals with either homozygous variants or compound heterozygous variants where one variant is known to be severe, correlated really well with the F-index of those variants (Pearson correlations of r(22)=0.6807, p = .000251) and r(40)=0.5269, p = .000337) respectively, Figure S2), it was decided to include these data as indicated in Table 2. Log10 and square root transformations were applied to obtain a normal distribution of the data, allowing to calculate the Pearson correlation (Supplemental Methods Table 2).

**Supplemental Methods Table 2 Cut-off values for functional study data**

*Only applied for synonymous and intronic variants

|  | **PS3_Moderate** | **PS3_Supporting** | **BS3_Supporting*** |
| --- | --- | --- | --- |
| **WT RNA %** | <20% | ≥20% and <50% | >80% |
| **F-index** | <0.15 | ≥0.15 and <0.50 | - |

After applying the steps PS1 *Same amino acid change*, PM5 *Novel missense at the same position* and PS3 BS3 *Functional studies*, the step PM3 BP2 *Cis/trans* Testing was executed in iteration until no further changes were observed.

**References**

1. Richards, S., et al., *Standards and guidelines for the interpretation of sequence variants: a joint consensus recommendation of the American College of Medical Genetics and Genomics and the Association for Molecular Pathology.* Genetics in medicine, 2015. **17**(5): p. 405-423.

2. *svi_criteria_nomenclature_recommendation_v1.pdf (clinicalgenome.org)*. 2017; Available from: https://clinicalgenome.org/site/assets/files/3459/svi_criteria_nomenclature_recommendation_v1.pdf.

3. Tavtigian, S.V., et al., *Fitting a naturally scaled point system to the ACMG/AMP variant classification guidelines.* Human mutation, 2020. **41**(10): p. 1734-1737.

4. Abou Tayoun, A.N., et al., *Recommendations for interpreting the loss of function PVS1 ACMG/AMP variant criterion.* Human mutation, 2018. **39**(11): p. 1517-1524.

5. Riepe, T.V., et al., *Benchmarking deep learning splice prediction tools using functional splice assays.* Human Mutation, 2021. **42**(7): p. 799-810.

6. Jaganathan, K., et al., *Predicting splicing from primary sequence with deep learning.* Cell, 2019. **176**(3): p. 535-548. e24.

7. Sangermano, R., et al., *ABCA4 midigenes reveal the full splice spectrum of all reported noncanonical splice site variants in Stargardt disease.* Genome Research, 2018. **28**(1): p. 100-110.

8. *VCF Submission - CI-SpliceAI*. Available from: https://ci-spliceai.com/.

9. Strauch, Y., et al., *CI-SpliceAI—Improving machine learning predictions of disease causing splicing variants using curated alternative splice sites.* Plos one, 2022. **17**(6): p. e0269159.

10. Wang, P., et al., *An ophthalmic targeted exome sequencing panel as a powerful tool to identify causative mutations in patients suspected of hereditary eye diseases.* Translational vision science & technology, 2019. **8**(2): p. 21-21.

11. Ghosh, R., et al., *Updated recommendation for the benign stand‐alone ACMG/AMP criterion.* Human mutation, 2018. **39**(11): p. 1525-1530.

12. *Frequency Filter (cardiodb.org)*. Available from: http://cardiodb.org/allelefrequencyapp/.

13. *pm2_-_svi_recommendation_-_approved_sept2020.pdf (clinicalgenome.org)*. 2020; Available from: https://clinicalgenome.org/site/assets/files/5182/pm2_-_svi_recommendation_-_approved_sept2020.pdf.

14. Cornelis, S.S., et al., *In silico functional meta‐analysis of 5,962 ABCA4 variants in 3,928 retinal dystrophy cases.* Human mutation, 2017. **38**(4): p. 400-408.

15. Karczewski, K.J., et al., *The mutational constraint spectrum quantified from variation in 141,456 humans.* Nature, 2020. **581**(7809): p. 434-443.

16. Lee, W., et al., *Cis-acting modifiers in the ABCA4 locus contribute to the penetrance of the major disease-causing variant in Stargardt disease.* Human molecular genetics, 2021. **30**(14): p. 1293-1304.

17. Cornelis, S.S., et al., *Personalized genetic counseling for Stargardt disease: Offspring risk estimates based on variant severity.* The American Journal of Human Genetics, 2022. **109**(3): p. 498-507.

18. Benjamini, Y. and Y. Hochberg, *Controlling the false discovery rate: a practical and powerful approach to multiple testing.* Journal of the Royal statistical society: series B (Methodological), 1995. **57**(1): p. 289-300.

19. Karolchik, D., et al., *The UCSC Table Browser data retrieval tool.* Nucleic acids research, 2004. **32**(suppl_1): p. D493-D496.

20. Karolchik, D., A.S. Hinrichs, and W.J. Kent, *The UCSC genome browser.* Current protocols in human genetics, 2011. **71**(1): p. 18.6. 1-18.6. 33.

21. Pejaver, V., et al., *Evidence-based calibration of computational tools for missense variant pathogenicity classification and ClinGen recommendations for clinical use of PP3/BP4 criteria.* bioRxiv, 2022.

22. Ioannidis, N.M., et al., *REVEL: an ensemble method for predicting the pathogenicity of rare missense variants.* The American Journal of Human Genetics, 2016. **99**(4): p. 877-885.

23. Rentzsch, P., et al., *CADD: predicting the deleteriousness of variants throughout the human genome.* Nucleic acids research, 2019. **47**(D1): p. D886-D894.

24. Kircher, M., et al., *A general framework for estimating the relative pathogenicity of human genetic variants.* Nature genetics, 2014. **46**(3): p. 310-315.

25. *HGVS variant name parsing and generation*. Available from: https://github.com/counsyl/hgvs.

26. Brnich, S.E., et al., *Recommendations for application of the functional evidence PS3/BS3 criterion using the ACMG/AMP sequence variant interpretation framework.* Genome medicine, 2020. **12**(1): p. 1-12.

27. Albert, S., et al., *Identification and rescue of splice defects caused by two neighboring deep-intronic ABCA4 mutations underlying Stargardt disease.* The American Journal of Human Genetics, 2018. **102**(4): p. 517-527.

28. Khan, M., et al., *Detailed phenotyping and therapeutic strategies for intronic ABCA4 variants in Stargardt disease.* Molecular Therapy-Nucleic Acids, 2020. **21**: p. 412-427.

29. Curtis, S.B., et al., *Functional analysis and classification of homozygous and hypomorphic ABCA4 variants associated with Stargardt macular degeneration.* Human mutation, 2020. **41**(11): p. 1944-1956.
