# Supplementary material for: Compendium of Clinical Variant Classification for 2,246 Unique ABCA4 Variants to Clarify Variant Pathogenicity in Stargardt Disease Using a Modified ACMG/AMP Framework
Source: Hum Mutat. 2023 Dec 26;2023:6815504. doi: 10.1155/2023/6815504 (PMC11918811; doi:10.1155/2023/6815504)
Supplement: Supplementary Materials — A file containing supplemental material pertinent to this manuscript is available. This supplemental information consists of 13 tables, 2 figures, and detailed methods, as follows: Table S1: all variants with ACMG/AMP classifications (column H) and severity subclassifications from [27] (column I). ACMG/AMP classifications are based on the point system as described by Tavtigian et al. [33]. In short, supporting, moderate, strong, and very strong evidence is combined into a score where each type of evidence gives a score of 1, 2, 4, or 8, respectively, where pathogenic evidence gives a positive score and benign evidence gives a negative score. The resulting total score per variant results in a benign (<-6), likely benign (-1–-6), VUS (0–5), likely pathogenic (6–9), or pathogenic (>9) classification. Table S2: ACMG/AMP classification step PVS1 Null variants. Table S3: ACMG/AMP classification step PM6 de novo variants. Table S4: ACMG/AMP classification step PS4 variant frequency and use of control populations. Table S5: ACMG/AMP classification step PM4 protein length changes due to in-frame deletions/insertions and stop losses. Table S6: ACMG/AMP classification steps PP3 and BP4 computational (in silico) data. Table S7: ACMG/AMP classification step BP7 synonymous variants. Table S8: ACMG/AMP classification steps BS1 and PM2 variant frequency and use of control populations. Table S9: ACMG/AMP classification steps PS1 and PM5 Same amino acid change and novel missense at the same position. Table S10: ACMG/AMP classification steps PS3 and BS3 functional studies. Table S11: three most frequent (likely) pathogenic variants per gnomAD population. Table S12: previously reported frequent pathogenic variants based on literature. Table S13: published segregating complex alleles. Figure S1: in silico comparison of CADD and REVEL for missense variants in ABCA4. In silico comparison of ABCA4 missense variants. CADD PHRED values are plotted against REVEL values. Cut-off values between [file 6815504.f1.zip › Table S12.docx]

**Table S12 Previously reported frequent Pathogenic variants based on literature**

| **DNA variant** | **Protein variant** | **ACMG/AMP classification** | **gnomAD allele frequency (GRCh37/hg19, v2.1.1) (population with highest frequency)** | **Region or population (reference)** |
| --- | --- | --- | --- | --- |
| c.768G>T | p.Leu257Valfs*17 | Pathogenic | 0.000186  (non-Finnish European) | Netherlands, [1] |
| c.859-9T>C | p.[=,Phe287_Arg452del] | Pathogenic | 0.000686  (South Asian) | American of South Asian descent, [2, 3] |
| c.[1622T>C;  3113C>T] | p.[(Leu541Pro);(Ala1038Val)] | Likely Pathogenic | Not applicable | Germany and Poland, [4-7] |
| c.2588G>C | p.[Gly863Ala,Gly863del] | Pathogenic | 0.00784  (Non-Finnish European) | Western Europe, [8, 9] |
| c.2894A>G | p.(Asn965Ser) | Pathogenic | 0.000434924  (East Asian) | Denmark and China, [10, 11] |
| c.3386G>T | p.(Arg1129Leu) | Pathogenic | 0.001665  (Latino/Admixed American) | Spain, [12] |
| c.4139C>T | p.(Pro1380Leu) | Pathogenic | 0.002318393  (Ashkenazi Jewish) | Ashkenazi Jewish, [13] |
| c.4254-37  _4254-15del | p.(?) | Not categorized | absent | Ashkenazi Jewish, [14] |
| c.4469G>A | p.(Cys1490Tyr) | Pathogenic | 0.00013  (non-Finnish European) | South African of European descent, [15] |
| c.4539+2001G>A | p.[=,Arg1514Leufs*36] | Pathogenic | 6.48E-05  (Non-Finnish European) | Belgium, [16-18] |
| c.5318C>T | p.(Ala1773Val) | Pathogenic | 0.000463  (Latino/Admixed American) | Mexico, [19, 20] |
| c.5882G>A | p.(Gly1961Glu) | Pathogenic | 0.023341049  (Ashkenazi Jewish) | Somalia and American of South Asian descent, [2, 21, 22] |
| c.5917del | p.(Val1973*) | Pathogenic | 0.000163  (South Asian) | Hungary and Bulgaria, [23, 24] |
| c.6320G>A | p.(Arg2107His) | Likely Pathogenic | 0.020794935  (African) | African American, [25] |

**References**

1. Cremers, F., et al. *The expanding roles of ABCA4 and CRB1 in inherited blindness*. in *Retinal Dystrophies: Functional Genomics to Gene Therapy: Novartis Foundation Symposium 255*. 2003. Wiley Online Library.

2. Lee, W., et al., *Genotypic spectrum and phenotype correlations of ABCA4-associated disease in patients of south Asian descent.* European Journal of Human Genetics, 2017. **25**(6): p. 735-743.

3. Zernant, J., et al., *Analysis of the ABCA4 genomic locus in Stargardt disease.* Human molecular genetics, 2014. **23**(25): p. 6797-6806.

4. Rivera, A., et al., *A comprehensive survey of sequence variation in the ABCA4 (ABCR) gene in Stargardt disease and age-related macular degeneration.* The American Journal of Human Genetics, 2000. **67**(4): p. 800-813.

5. Ścieżyńska, A., et al., *Next-generation sequencing of ABCA4: High frequency of complex alleles and novel mutations in patients with retinal dystrophies from Central Europe.* Experimental eye research, 2016. **145**: p. 93-99.

6. Tracewska, A.M., et al., *Genetic spectrum of ABCA4-associated retinal degeneration in poland.* Genes, 2019. **10**(12): p. 959.

7. Zolnikova, I.V., et al., *Stargardt disease-associated mutation spectrum of a Russian Federation cohort.* European Journal of Medical Genetics, 2017. **60**(2): p. 140-147.

8. Maugeri, A., et al., *The 2588G→ C mutation in the ABCR gene is a mild frequent founder mutation in the Western European population and allows the classification of ABCR mutations in patients with Stargardt disease.* The American Journal of Human Genetics, 1999. **64**(4): p. 1024-1035.

9. Maugeri, A., et al., *The ABCA4 2588G> C Stargardt mutation: single origin and increasing frequency from South-West to North-East Europe.* European Journal of Human Genetics, 2002. **10**(3): p. 197-203.

10. Rosenberg, T., et al., *N965S is a common ABCA4 variant in Stargardt-related retinopathies in the Danish population.* Mol Vis, 2007. **13**: p. 1962-1969.

11. Jiang, F., et al., *Screening of ABCA4 gene in a Chinese cohort with Stargardt disease or cone-rod dystrophy with a report on 85 novel mutations.* Investigative Ophthalmology & Visual Science, 2016. **57**(1): p. 145-152.

12. Valverde, D., et al., *Microarray-based mutation analysis of the ABCA4 gene in Spanish patients with Stargardt disease: evidence of a prevalent mutated allele.* Mol Vis, 2006. **12**: p. 902-908.

13. Sharon, D., et al., *A nationwide genetic analysis of inherited retinal diseases in Israel as assessed by the Israeli inherited retinal disease consortium (IIRDC).* Human mutation, 2020. **41**(1): p. 140-149.

14. Beit-Ya'acov, A., et al., *Homozygosity for a novel ABCA4 founder splicing mutation is associated with progressive and severe Stargardt-like disease.* Investigative ophthalmology & visual science, 2007. **48**(9): p. 4308-4314.

15. September, A.V., et al., *Mutation spectrum and founder chromosomes for the ABCA4 gene in South African patients with Stargardt disease.* Investigative ophthalmology & visual science, 2004. **45**(6): p. 1705-1711.

16. Bauwens, M., et al., *An Augmented ABCA 4 Screen Targeting Noncoding Regions Reveals a Deep Intronic Founder Variant in B elgian S targardt Patients.* Human mutation, 2015. **36**(1): p. 39-42.

17. Bauwens, M., et al., *ABCA4-associated disease as a model for missing heritability in autosomal recessive disorders: novel noncoding splice, cis-regulatory, structural, and recurrent hypomorphic variants.* Genetics in Medicine, 2019. **21**(8): p. 1761-1771.

18. Bax, N.M., et al., *Heterozygous Deep‐Intronic Variants and Deletions in ABCA 4 in Persons with Retinal Dystrophies and One Exonic ABCA 4 Variant.* Human mutation, 2015. **36**(1): p. 43-47.

19. Chacón-Camacho, O.F., et al., *ABCA4 mutational spectrum in Mexican patients with Stargardt disease: identification of 12 novel mutations and evidence of a founder effect for the common p. A1773V mutation.* Experimental eye research, 2013. **109**: p. 77-82.

20. López-Rubio, S., et al., *Retinal phenotypic characterization of patients with ABCA4 retinopathydue to the homozygous p. Ala1773Val mutation.* Molecular Vision, 2018. **24**: p. 105.

21. Guymer, R.H., et al., *Variation of codons 1961 and 2177 of the Stargardt disease gene is not associated with age-related macular degeneration.* Archives of ophthalmology, 2001. **119**(5): p. 745-751.

22. Burke, T.R., et al., *Retinal phenotypes in patients homozygous for the G1961E mutation in the ABCA4 gene.* Investigative ophthalmology & visual science, 2012. **53**(8): p. 4458-4467.

23. Hargitai, J., et al., *Correlation of clinical and genetic findings in Hungarian patients with Stargardt disease.* Investigative ophthalmology & visual science, 2005. **46**(12): p. 4402-4408.

24. Kamenarova, K., et al., *Panel‐based next‐generation sequencing identifies novel mutations in Bulgarian patients with inherited retinal dystrophies.* Molecular Genetics & Genomic Medicine, 2022: p. e1997.

25. Zernant, J., et al., *Genetic and Clinical Analysis of ABCA 4‐Associated Disease in African American Patients.* Human mutation, 2014. **35**(10): p. 1187-1194.
