# Supplementary figures and images for: Compendium of Clinical Variant Classification for 2,246 Unique ABCA4 Variants to Clarify Variant Pathogenicity in Stargardt Disease Using a Modified ACMG/AMP Framework
Source: Hum Mutat. 2023 Dec 26;2023:6815504. doi: 10.1155/2023/6815504 (PMC11918811; doi:10.1155/2023/6815504)

## Slide 1
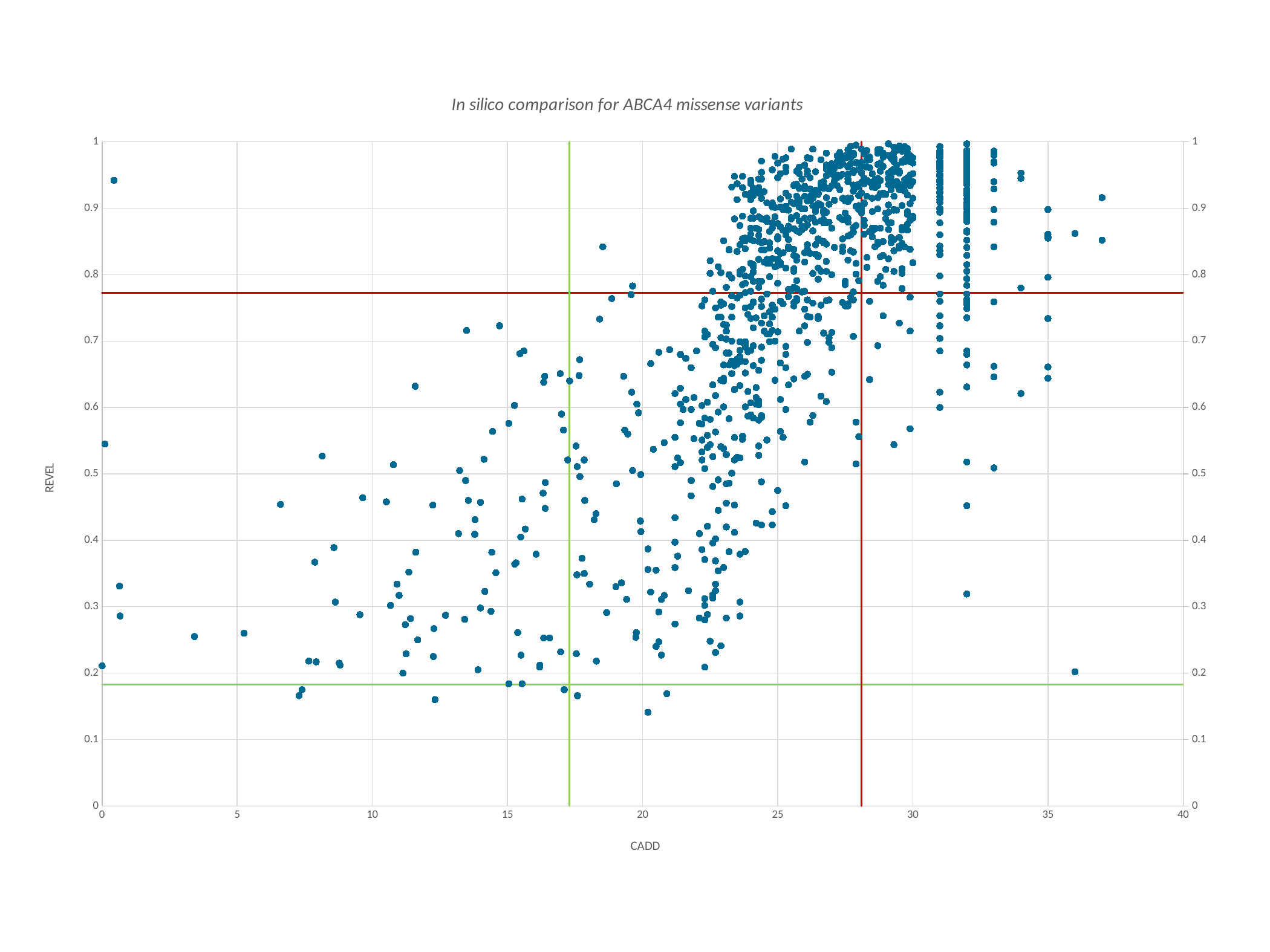

### Chart: In silico comparison for ABCA4 missense variants
| Category | REVEL | | | | | |
|---|---|---|---|---|---|---|

Supplement: Supplementary Materials — A file containing supplemental material pertinent to this manuscript is available. This supplemental information consists of 13 tables, 2 figures, and detailed methods, as follows: Table S1: all variants with ACMG/AMP classifications (column H) and severity subclassifications from [27] (column I). ACMG/AMP classifications are based on the point system as described by Tavtigian et al. [33]. In short, supporting, moderate, strong, and very strong evidence is combined into a score where each type of evidence gives a score of 1, 2, 4, or 8, respectively, where pathogenic evidence gives a positive score and benign evidence gives a negative score. The resulting total score per variant results in a benign (<-6), likely benign (-1–-6), VUS (0–5), likely pathogenic (6–9), or pathogenic (>9) classification. Table S2: ACMG/AMP classification step PVS1 Null variants. Table S3: ACMG/AMP classification step PM6 de novo variants. Table S4: ACMG/AMP classification step PS4 variant frequency and use of control populations. Table S5: ACMG/AMP classification step PM4 protein length changes due to in-frame deletions/insertions and stop losses. Table S6: ACMG/AMP classification steps PP3 and BP4 computational (in silico) data. Table S7: ACMG/AMP classification step BP7 synonymous variants. Table S8: ACMG/AMP classification steps BS1 and PM2 variant frequency and use of control populations. Table S9: ACMG/AMP classification steps PS1 and PM5 Same amino acid change and novel missense at the same position. Table S10: ACMG/AMP classification steps PS3 and BS3 functional studies. Table S11: three most frequent (likely) pathogenic variants per gnomAD population. Table S12: previously reported frequent pathogenic variants based on literature. Table S13: published segregating complex alleles. Figure S1: in silico comparison of CADD and REVEL for missense variants in ABCA4. In silico comparison of ABCA4 missense variants. CADD PHRED values are plotted against REVEL values. Cut-off values between [file 6815504.f1.zip › Figure S1.pptx]
